# Age and the means of bypassing stasis influence the intrinsic subtype of immortalized human mammary epithelial cells

**DOI:** 10.3389/fcell.2015.00013

**Published:** 2015-03-11

**Authors:** Jonathan K. Lee, James C. Garbe, Lukas Vrba, Masaru Miyano, Bernard W. Futscher, Martha R. Stampfer, Mark A. LaBarge

**Affiliations:** ^1^Life Sciences Division, Lawrence Berkeley National LaboratoryBerkeley, CA, USA; ^2^Arizona Cancer Center, The University of ArizonaTucson, AZ, USA; ^3^College of Pharmacy, Department of Pharmacology and Toxicology, The University of ArizonaTucson, AZ, USA

**Keywords:** human mammary epithelial cell, HMEC, breast cancer, immortalization, aging, stasis, intrinsic subtype

## Abstract

Based on molecular features, breast cancers are grouped into intrinsic subtypes that have different prognoses and therapeutic response profiles. With increasing age, breast cancer incidence increases, with hormone receptor-positive and other luminal-like subtype tumors comprising a majority of cases. It is not known at what stage of tumor progression subtype specification occurs, nor how the process of aging affects the intrinsic subtype. We examined subtype markers in immortalized human mammary epithelial cell lines established following exposure of primary cultured cell strains to a two-step immortalization protocol that targets the two main barriers to immortality: stasis (stress-associated senescence) and replicative senescence. Cell lines derived from epithelial cells obtained from non-tumorous pre- and post-menopausal breast surgery tissues were compared. Additionally, comparisons were made between lines generated using two different genetic interventions to bypass stasis: transduction of either an shRNA that down-regulated p16^INK4A^, or overexpressed constitutive active cyclin D1/CDK2. In all cases, the replicative senescence barrier was bypassed by transduction of c-Myc. Cells from all resulting immortal lines exhibited normal karyotypes. Immunofluorescence, flow cytometry, and gene expression analyses of lineage-specific markers were used to categorize the intrinsic subtypes of the immortalized lines. Bypassing stasis with p16 shRNA in young strains generated cell lines that were invariably basal-like, but the lines examined from older strains exhibited some luminal features such as keratin 19 and estrogen receptor expression. Overexpression of cyclin D1/CDK2 resulted in keratin 19 positive, luminal-like cell lines from both young and old strains, and the lines examined from older strains exhibited estrogen receptor expression. Thus age and the method of bypassing stasis independently influence the subtype of immortalized human mammary epithelial cells.

## Introduction

Different patterns of gene and protein expression in breast tumors have led to the delineation of at least four intrinsic subtypes of breast cancers: HER2-related, luminal A, luminal B, and basal-like (Perou et al., 2000). To some extent, the subtype designations depend on their relative molecular similarity to normal luminal and basal lineages of the mammary epithelium. The incidence of breast cancer not only increases with age, but tumors with luminal intrinsic subtypes are strikingly over-represented among post-menopausal patients (Jenkins et al., 2014). Luminal subtype tumors, compared to basal-types, have the lowest mutation rates and yet they exhibit the greatest diversity of somatic mutations (Cancer Genome Atlas Network, 2012), suggesting that early events in cancer progression are key to establishing that subtype. Here we interrogate the impacts of chronological age and the earliest molecular events in malignant progression, the means of bypassing stasis, on the intrinsic subtype of immortalized human mammary epithelial cells (HMEC).

In order for normal HMEC to give rise to malignancies they must bypass a number of rate-limiting tumor-suppressive senescence barriers: stress-associated stasis, replicative senescence, and oncogene-induced senescence (Stampfer et al., 2013). Because stasis is enforced by active retinoblastoma (RB), silencing or mutation of p16, over-expression of cyclin D1, or other errors in the RB pathway, can bypass this barrier. Overexpression of cyclin D1 is frequently observed in luminal subtype breast tumors, whereas p16 is down-modulated by a number of epigenetic and genetic mechanisms and the subtype association is less pronounced (Cancer Genome Atlas Network, 2012).

The process of aging is associated with distinctive changes in the patterns of gene expression in human tissues (Rodwell et al., 2004; Zahn et al., 2006; Garbe et al., 2012), but there is little understanding of how these changes relate to breast cancer susceptibility or to cancer intrinsic subtypes. We have previously shown that functional, molecular, and biochemical hallmarks of mammary epithelial lineages, and of chronological age *in vivo*, are preserved in early passage cultures of primary normal finite lifespan pre-stasis HMEC (Garbe et al., 2012). Therefore age-related transcriptome changes are metastable through unknown mechanisms and functional consequences of aging at the cellular level can be studied using the proper culture systems. Normal pre-stasis HMEC can be efficiently immortalized in a two-step process of targeted genetic modifications that first bypasses the stasis barrier and then trans-activates telomerase (Garbe et al., 2014). The resultant non-clonal immortalized lines have normal karyotypes and lack the confounding gross genomic changes commonly seen in tumors, tumor-derived cell lines, and clonally derived *in vitro* immortalized lines. Here we used this method of immortalization of normal HMEC from four individuals who represent a range of chronological age as a platform to assess the outcome of breast cancer-relevant changes in age-relevant backgrounds. We found that chronological age and the method of bypassing stasis independently influences the intrinsic subtype in immortalized HMEC.

## Materials and methods

### Cell culture

Finite lifespan HMEC from specimens 184 batch D, 240L batch B, 122L, and 805P were obtained from reduction mammoplasty tissue or normal tissue peripheral to a tumor (805P) from women aged 21, 19, 66, and 91 years, respectively, and were cultured as pre-stasis strains (Labarge et al., 2013). Pre-stasis, post-stasis, and immortalized HMEC were grown in M87A medium supplemented with cholera toxin (CT) at 0.5 ng/ml, and oxytocin (X) at 0.1 nM (Garbe et al., 2009). Post-selection post-stasis HMEC 184 batch B were grown in serum-free MCDB 170 medium (MEGM basal (without the bullet kit), Lonza, Walkersville, MD) plus supplements. Total population doublings (PD) were calculated as described (Garbe et al., 2009). 3-D cultures were conducted as embedded Matrigel cultures for 14 days in M87A+CT+X. Karyology was performed by Roswell Park Cancer Institute SKY/FISH Facility (Buffalo, NY).

### Retroviral transduction

The p16 shRNA vector (MSCV) was obtained from Greg Hannon. The pBabe-hygro-myc was used to transduce cells with c-Myc. The WPI-Cyclin D1/CDK2-GFP retrovirus was obtained from Mark Jackson. Retroviral containing supernatants were collected in M87A medium and transductions were performed as described (Garbe et al., 2014).

### Immunofluorescence

HMEC were fixed in methanol:acetone (1:1) at −20°C for 15 min, blocked with PBS, 5% normal goat serum, 0.1% Triton X-100, and incubated with K14 (Covance #PRB-155P-100, polyclonal rabbit, 1:1000) and K19 (Developmental Studies Hybridoma Bank, clone Troma-III, 1:20) overnight at 4°C, then visualized with fluorescent secondary antibodies (Invitrogen), and incubated for 2 h at room temperature. For 3-D cultures, HMEC were fixed in 4% paraformaldehyde for 30 min, blocked with PBS, 5% normal goat serum, 0.1% Triton X-100, and incubated with β-Catenin (BD Transduction Laboratories #610154, clone 14/Beta-Catenin, Mouse IgG1, 1:200) and Estrogen Receptor-α (Abcam #ab16660, clone SP1, rabbit monoclonal, 1:100) overnight at 4°C, then with secondaries for 2 h at room temp.

### Flow cytometry

CD227-FITC (BD Bioscience #559774, clone HMPV, 1:50), CD10-PE (BioLegend #312204, clone HI10a, 1:100), CD10-APC (BioLegend #312210, clone HI10a, 1:100), CD227-PE (BioLegend #355604, clone 16A, 1:100) were added to cells in media for 25 min on ice, washed in PBS and analyzed with a FACSCalibur (Becton Dickinson).

### Western blot

The following antibodies were used: p16^INK4^ (BD Pharmigen #551154 Mouse 1:1000), Cyclin D1 (Santa Cruz Biotech #SC-246, clone HD11, mouse 1:200), beta-actin (Abcam #ab49900, Mouse 1:500), and visualized with goat anti mouse IgG Fc (HRP) conjugate (Abcam #ab97265, 1:5000).

### Microarray analysis

Subconfluent cultures were harvested for RNA 24 h following feeding. Total RNA was harvested using TRIzol and purified using the miRNeasy Kit (Qiagen). RNA labeling and hybridization to Affymetrix Human Gene 1.0 ST Arrays, was performed according to the manufacturer's protocols. Microarray data were analyzed in R programming environment using the limma package.

## Results

### Generation of immortal HMEC lines with p16sh RNA or cyclin D1/CDK2, and c-Myc

**Figure 1:**
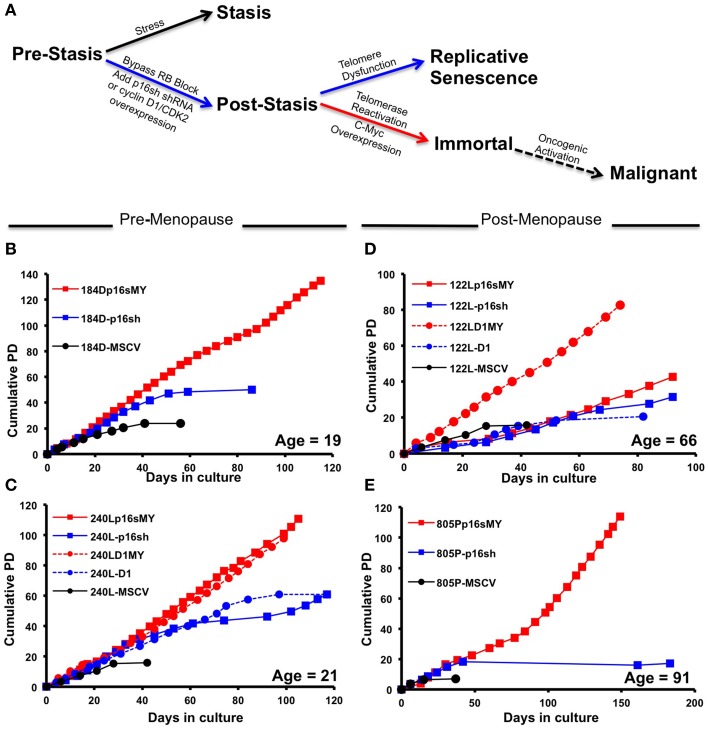
**Growth characterization of pre-stasis, post-stasis, and non-malignant immortally transformed HMEC. (A)** The stages of malignant progression in breast epithelia. **(B–E)** Growth curves starting from passage 4 plotted as population doublings vs. days in culture for strains and cell lines derived from specimen **(B)** 184, **(C)** 240L, **(D)** 122L, and **(E)** 805P. Each point indicates a passage. The chronological age of the patient at the time of surgery is indicated in the lower right corner of each plot.

To determine whether chronological age and early events in cancer progression are determinants of the intrinsic subtype of immortal HMEC, a two step procedure that does not cause gross genomic changes (Garbe et al., 2014) was used to immortalize primary pre-stasis HMEC from four women aged 19, 21, 66, and 91 years. Figure 1A illustrates the steps taken to bypass the stasis and immortalization barriers. Pre-stasis HMEC strains 184D, 240L, 122L, and 805P at 3rd passage (3p) were transduced with retroviruses that expressed either p16 shRNA (p16sh) or cyclin D1/CDK2 (D1), or with a control empty vector. These gave rise to the post-stasis cultures 184D-p16sh, 240L-p16sh, 240L-D1, 122L-p16sh, 122L-D1, and 805P-p16sh. Post-stasis strains were transduced at 4p either with an empty vector control, or a c-Myc expressing retrovirus in order to transactivate telomerase, giving rise to the non-clonal immortal lines 184Dp16sMY, 240Lp16sMY, 240LD1MY, 122Lp16sMY, 122LD1MY, and 805Pp16sMY. The pre-stasis cultures eventually ceased proliferation due to p16-mediated stasis, and post-stasis cultures ceased net proliferation due to telomere attrition-mediated telomere dysfunction. The immortal cell lines have continued to grow for over 25 passages (Figures 1B–E).

**Figure 2:**
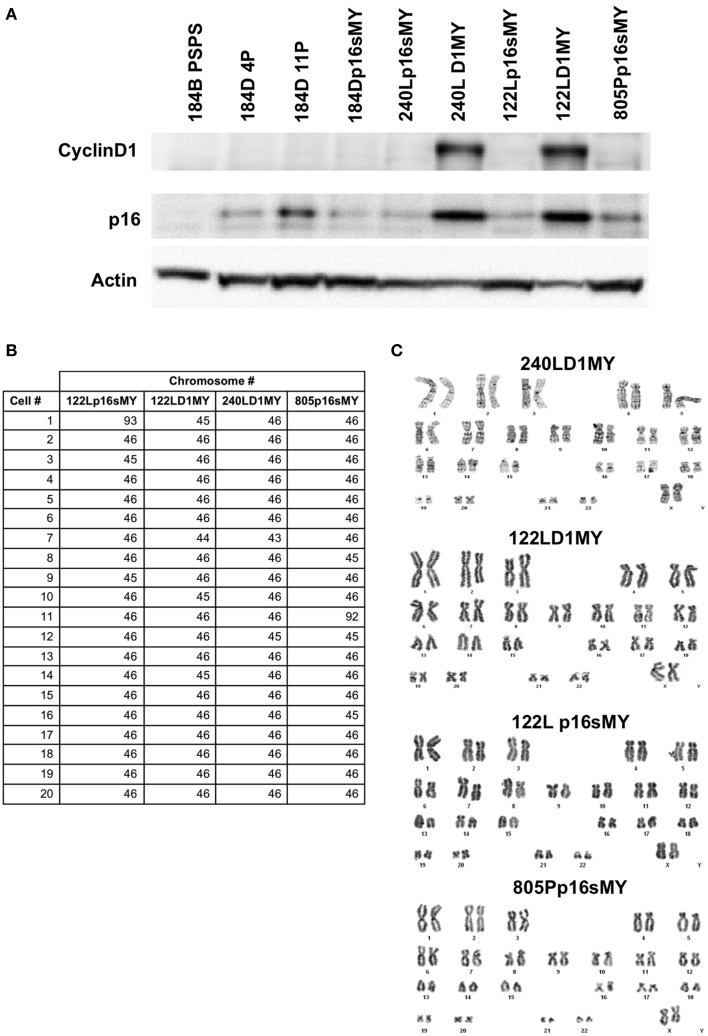
**Biochemical and karyologic characterization of non-malignant immortal HMEC cell lines. (A)** Western blot analysis of cyclinD1 and p16 protein levels in cell lysates. **(B)** Table of karyotypes of 20 cells each of the indicated cell lines. **(C)** Representative examples of karyotypes from the indicated cell lines.

Western blots were used to confirm knockdown of the p16 protein in the p16sh silenced cell lines, with 184B post-stasis post-selection cells, which are known to have silenced p16, as a control (Figure 2A). As expected, p16 protein levels increased as a function of passage in the control pre-stasis strains (Garbe et al., 2009), while levels were greatly reduced in the cultures transduced with p16sh. Cyclin D1 was abundant in the D1MY immortal cell lines, but was not over-represented in any of the other cell lines (Figure 2A). There also was abundant expression of p16 protein in the D1MY lines. We previously reported that karyotypes for early passage 184Dp16sMY and 240Lp16sMY were normal (Garbe et al., 2014), and karyotype analysis of metaphase chromosomes in early passage 240LD1MY, 122LD1MY, 122Lp16sMY, and 805Pp16sMY also were normal diploid (Figures 2B,C).

### Biochemical phenotypes of the immortal cell lines show intrinsic lineage biases

**Figure 3:**
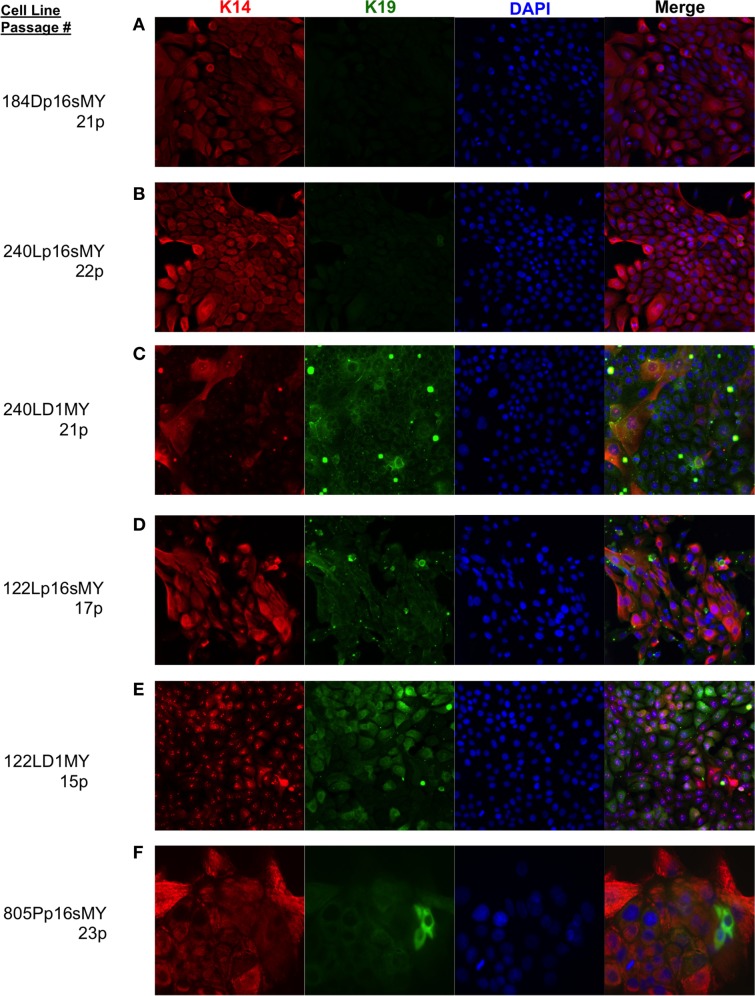
**Lineage-specific keratin protein expression in non-malignant immortal HMEC on 2-D culture substrata**. Representative immunofluorescence images showing keratin (K)14 (red) and K19 (green) expression in **(A)** 184Dp16sMY, **(B)** 240Lp16sMY, **(C)** 240LD1MY, **(D)** 122Lp16sMY, **(E)** 122LD1MY, and **(F)** 805Pp16sMY. Nuclei appear blue, bar represents 50 um.

**Figure 4:**
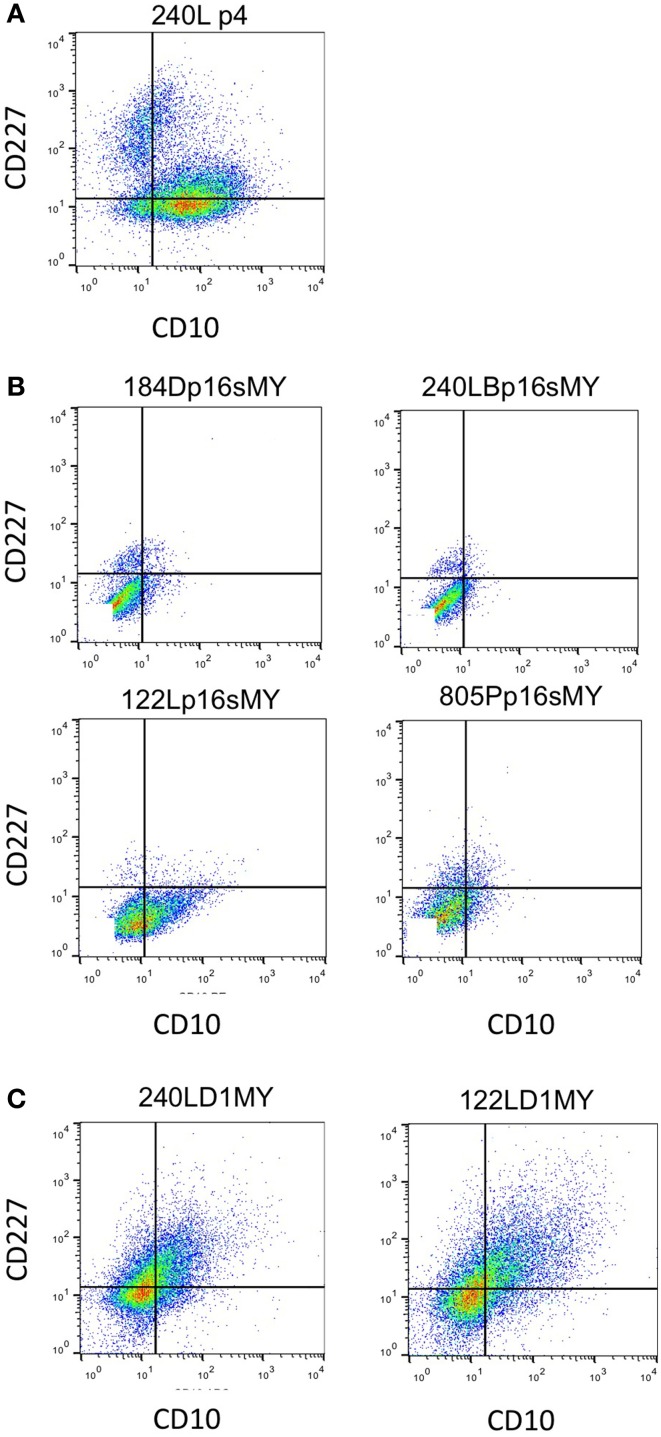
**Expression of cell surface based lineage markers**. Expression of CD227 and CD10 in **(A)** 4p normal pre-stasis finite strain 240L showing two clear populations CD10−/CD227+ luminal and CD10+/CD227− myoepithelial cells. **(B)** All four immortal non-malignant HMEC cell lines that used p16shRNA to bypass stasis. **(C)** Both cell lines that used cyclinD1/CDK2 to bypass stasis. Quadrant lines are indicated for frame of reference.

The immortalized lines were evaluated for expression of luminal and myoepithelial/basal lineage-specific proteins and gene transcripts to determine whether their intrinsic subtypes were more luminal- or basal-like. Keratin (K)14 and CALLA/CD10 are proteins expressed primarily in myoepithelia, and K19 and Sialomucin-1/CD227 in luminal epithelia (Villadsen et al., 2007). The two lines derived from the younger women (specimens 184 and 240L) following transduction of p16sh and c-Myc expressed only K14 when grown on 2-D tissue culture plastic (Figures 3A,B). The two lines derived from the older women (specimens 122L and 805P) exhibited mixtures of cells expressing K14, K19, and K14/K19 when exposed to the same p16sh and c-Myc protocol (Figures 3D,F). Both lines derived using cyclin D1/CDK2 to bypass stasis, 240LD1MY from a young woman and 122LD1MY from an older woman, exhibited significant K19 expression, both in cells expressing only K19 as well as expressing K14 and K19 (Figures 3C,E). Flow cytometry (FACS) analysis of CD10 and CD227 expression in normal pre-stasis 240L at 4p shows characteristically distinct CD227+/CD10− luminal and CD227−/CD10+ myoepithelial populations (Figure 4A). All of the p16sMY cell lines had only minor CD227+/CD10− luminal-like populations and generally low CD10 expression, with the exception of 122Lp16sMY that had more CD10 expression relative to the other cell lines (Figure 4B). In contrast, most of the cells of the two D1MY lines were CD227+/CD10+ (Figure 4C).

**Figure 5:**
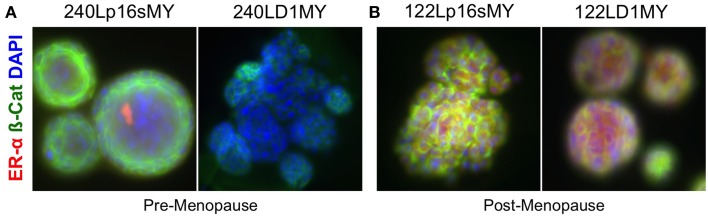
**Estrogen receptor and beta-catenin expression in 3-D**. Representative immunofluorescence images ERa (red) and B-catenin (green) expression in **(A)** 240p16sMY and D1MY, and **(B)** 122Lp16sMY and D1MY. Nuclei appear blue, bar represents 50 um.

To further assess the differential effects of age and of method of stasis bypass, expression of the estrogen receptor alpha (ERα) protein was evaluated in the p16sMY and D1MY cell lines derived from the young strain 240L and the older strain 122L. ERα is expressed by a subset of luminal cells in the normal mammary gland, and is used to categorize the luminal subtypes of breast cancers. The p16sMY and D1MY lines derived from 240L and 122L were cultured for 14 days embedded in 3-D Matrigel. ERα was not detected in either the p16sMY or D1MY line derived from 240L (Figure 5A), but it was richly expressed in both 122L-derived lines independent of the method used to bypass stasis (Figure 5B).

Altogether these data indicate that these non-clonal lines are heterogeneous, containing a mixture of lineages. However, both IF and FACS-based immune-phenotypes show an age-dependent increased expression of luminal markers in the lines that bypassed stasis with p16sh. Lines that bypassed stasis with cyclin D1/CDK2 overexpression generated a more luminal phenotype independent of age.

### Lineage-specific gene expression analysis of post-stasis and immortalized cell lines

Two sets of genes, specifically expressed in luminal or myoepithelial/basal cells, were identified based on microarray data from FACS enriched CD10+/CD227− myoepithelial and CD10−/CD227+ luminal epithelial 240L and 122L passage 4 pre-stasis cells using a cutoff of 2 fold gene expression enrichment in respective cell lineage relative to the other. The expression of these two lineage-specific sets of genes was then analyzed in the immortal p16sMY and D1MY cell lines, as well as in the post-stasis direct precursors to the immortal lines, which had been transduced with either p16 shRNA or cyclin D1 only.

**Figure 6:**
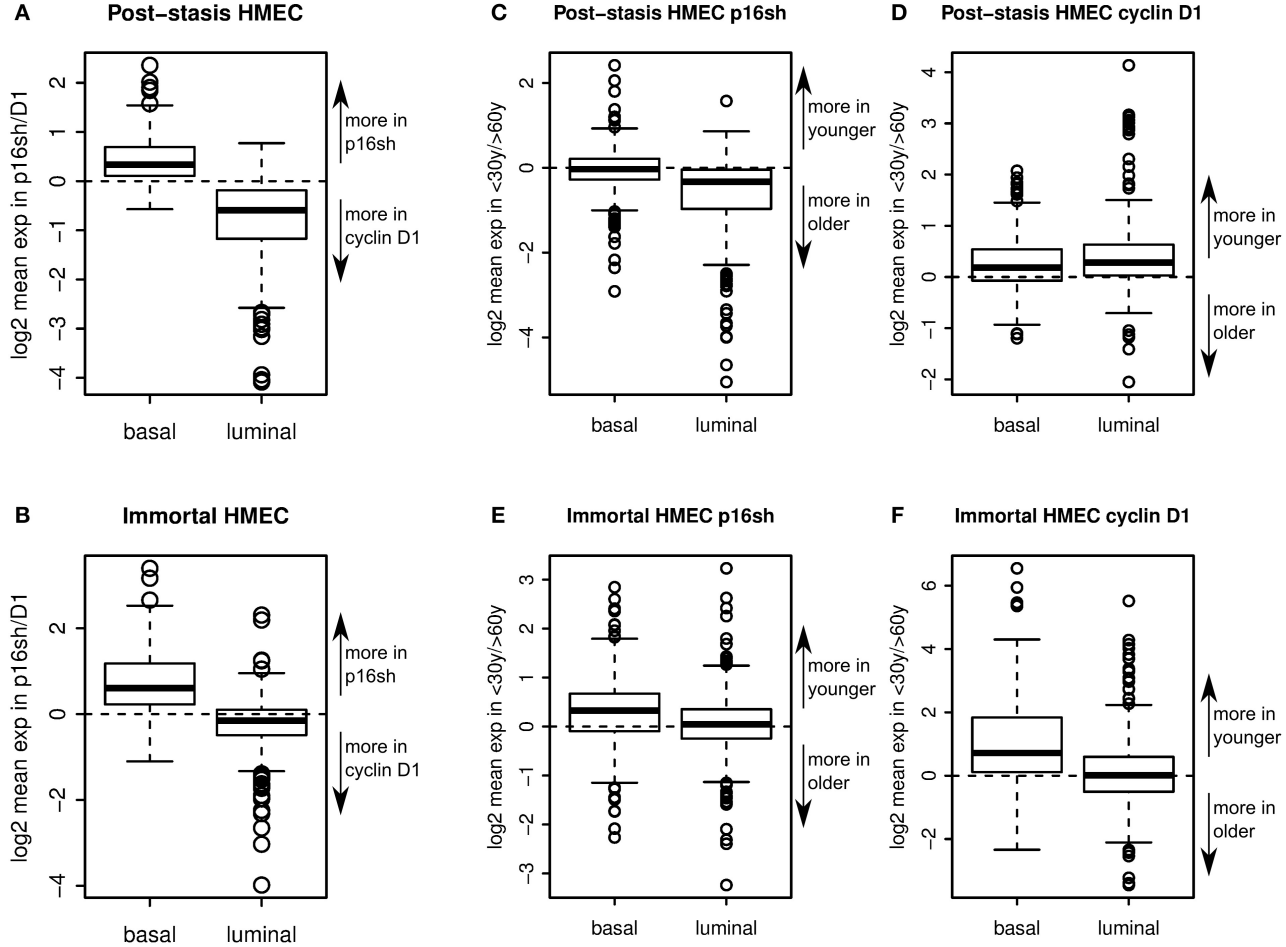
**Lineage-specific gene expression**. Boxplots showing enrichment of relative expression of 302 basal specific and 337 luminal specific transcripts in p16sh vs. D1 post-stasis HMEC **(A)**, and in p16sMY vs. D1MY immortal HMEC **(B)**. Boxplots showing enrichment of relative expression of luminal and basal specific transcripts in young vs. old donors in p16sh **(C)** and D1 cells **(D)** in post-stasis HMEC and in p16sh **(E)** and D1 **(F)** cells in immortal HMEC.

Comparison of gene expression in p16sh vs. D1 post-stasis cells showed that basal genes were expressed more in p16sh cells, whereas luminal genes were expressed more in D1 cells (Figure 6A). The trend was maintained in p16sMY vs. D1MY immortal cell lines (Figure 6B), suggesting that the means of bypassing stasis is a key step in establishing luminal-like or basal-like gene expression patterns. Post-stasis cells had a larger difference in luminal specific genes, while immortal cells differed more in basal specific genes (Figures 6A,B). The effect of age on the expression of these lineage related genes was also evident, although less pronounced. Comparing the ratios of the genes expressed in young vs. old post-stasis (Figures 6C,D) and immortal (Figures 6E,F) cultures showed overall preferential expression of basal compared to luminal markers in samples from younger vs. older donors. The basal genes were overexpressed in younger donors in D1 post-stasis and in immortal samples (Figures 6D–F). Similar to the means of bypassing the stasis, the differences for basal genes between ages were larger in the immortal cells (Figures 6E,F). The luminal specific genes were under-expressed in younger donors in p16sh cells (Figure 6C). In immortal cells (Figures 6E,F) there was no obvious bias towards older donors for luminal genes. Interestingly, in D1 post-stasis samples the ratio of luminal specific genes between ages was reversed (Figure 6C), suggesting a dominant effect of the means bypassing the stasis over the age of donor in case of D1, and stronger response in the younger donors with higher plasticity. Overall, overexpression of cyclin D1 to bypass stasis is associated with a luminal-like gene expression pattern, and knock down of p16 is associated with a basal-like gene expression pattern. The effect of age is also obvious, although it seems to be dominated by the means of bypassing the stasis. Both, the effect of bypassing the stasis and the age of donor, are concordant with the biochemical analyses in previous figures.

## Discussion

**Figure 7:**
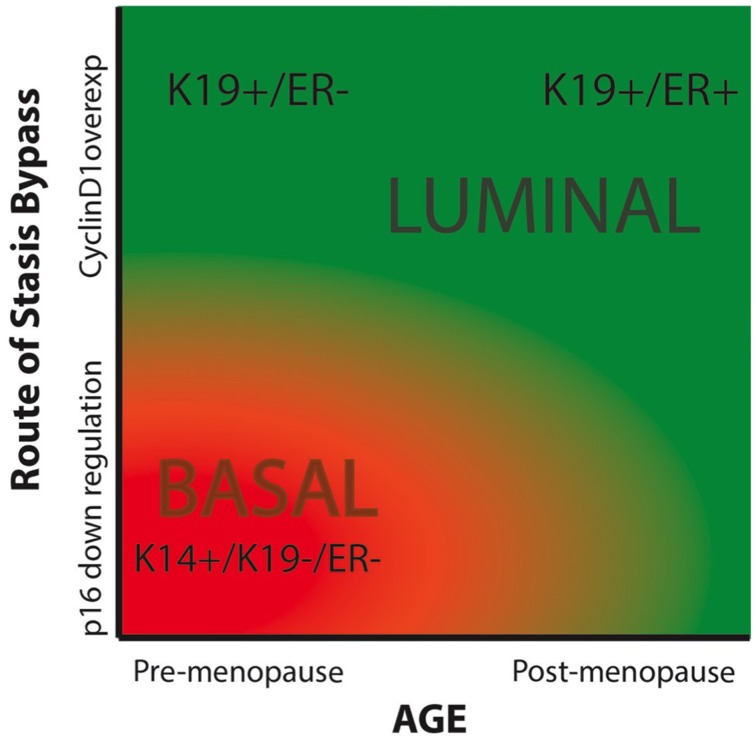
**Phase diagram summarizing our hypothesis of the impacts of chronological age and the means of bypassing stasis on the intrinsic subtype of immortal HMEC**. The color red indicated basal-like subtypes, and green represents luminal-like subtypes.

Breast cancers have been categorized by lineage markers into intrinsic subtypes that differ in prognosis and response to treatment. The mechanisms responsible for determining subtype have not been clearly defined; cell of origin, specific oncogenic insults, and cellular microenvironment have been proposed to influence lineage expression in cancer cells (Sims et al., 2007; Prat and Perou, 2011). However, lineage specificity in immortal and malignantly transformed cells is neither exact nor obvious by comparison to the normal lineages *in vivo*—they are caricatures of normal at the best. Indeed, every cell line had some level of heterogeneity, with varying distributions of cells representing the luminal and myoepithelial lineages. Here we have shown that expression of lineage-related markers in immortalized HMEC is influenced by both chronological age and the method by which the normal HMEC escape an initial tumor-suppressive senescence barrier, stress-associated stasis. A comparison of HMEC from young (<30 years) vs. older (>60 years) women showed that increased age biased toward generation of immortalized lines with greater expression of luminal phenotypes. A comparison of immortalized lines generated by using either p16sh or cyclin D1/CDK2 to bypass stasis showed a bias toward a luminal phenotype when cyclin D1/CDK2 was utilized, independent of age. Advanced age combined with cyclin D1-mediated bypass of stasis generated a line that was qualitatively the most luminal, exhibiting expression of K19, CD227, ERα, and other luminal transcripts. While a younger strain transduced with cyclin D1 also gave rise to a K19 and CD227-expressing luminal line, it did not produce ERα. In contrast, the younger lines that bypassed stasis with p16sh were qualitatively very basal-like (K19−/K14+, CD227− and basal transcripts). These results are based on our currently small number of cell lines, due in part to the length of time required to generate and examine each line. As more immortal cell lines are generated using this methodology the relative importance of age and stasis bypass in the etiology of intrinsic subtypes will become more obvious. Using a phase diagram we have expressed our hypothesis that the relative luminal vs. basal phenotype of immortalized HMEC is influenced by both chronological age and the method of stasis bypass (Figure 7).

A strength of our approach is that the outcome of the immortalization process can be evaluated in the absence of confounding gene mutations and gross genomic re-arrangements (Garbe et al., 2014). Thus it is reasonable to assume a given targeted genetic change played a role in the final phenotypes of the cell lines that were generated. By comparison, the diverse collection of tumor-derived breast cancer cell lines available bear a large number of genetic and epigenetic changes, making it difficult to causally link specific changes to an intrinsic subtype, or indeed to the process of becoming immortal (Neve et al., 2006). Here we were able to control two variables, age and the method of bypassing stasis barrier, while holding other variables constant, such the use of c-Myc to transactivate telomerase to bypass replicative senescence. Our results thus implicate the earliest events in cancer progression—chronological age of the cell of origin and the molecular pathway used to bypass stasis—as key determinants of breast cancer subtype.

These targeted genetic approaches to bypass the stasis barrier and replicative senescence convert a large percentage of the initial pre-stasis HMEC population into immortal cells, generating non-clonal immortalized lines. Stasis in normal HMEC has been bypassed by a variety of oncogenic exposures such as the chemical carcinogen benzo(a)pyrene (Stampfer and Bartley, 1985), inactivation of p53 (Garbe et al., 2007), and transduced c-Myc (Garbe et al., 2014). However in all these situations, unlike the genetic targeting employed here, the errors occured in single cells, yielding clonal post-stasis cultures. Stasis has also been overcome when HMEC are grown in high stress defined media, such as the commonly used, commercially sold, MCDB 170-type, MEGM and M171 media (Hammond et al., 1984; Brenner et al., 1998). The resultant post-selection post-stasis HMEC, such as 184B PSPS and many of the “normal HMEC” sold commercially, have an abnormal phenotype and may represent the precursor to metaplastic breast cancers (Keller et al., 2012; Sauder et al., 2014). Post-selection strains are not suited to the type of analysis presented here as in addition to containing many alterations in gene expression and epigenetic marks compared to normal HMEC, or cultures made post-stasis by direct targeting, they are not immortalized by transduced c-Myc (Li et al., 2007; Garbe et al., 2009, 2014; Novak et al., 2009).

Other factors that still need to be considered in our conceptual framework of intrinsic subtypes are the potential impacts of the initial lineage of the transformed cells, transduction bias, and the persistence of the phenotypes in immortal non-malignant cells as they progress to malignancy. In one hallmark study of mammary tumor-type etiology, primary epithelia from breast reductions were dissociated then transduced with lentiviral vectors that expressed either (i) mutant p53, cyclin D1, K-ras, and myristolated PI3K, or (ii) SV40 and K-ras, then were sorted into EpCAM+ or CD10+ subpopulations prior to orthotopic injection into mouse hosts (Keller et al., 2012). These experiments showed that the EpCAM+ cells could give rise to both luminal and basal tumors, whereas the CD10+ cells only gave rise to metaplastic tumors. Although effective at causing malignant transformation, neither of the transformation gene cocktails represent changes that occur frequently in breast cancer (particularly the SV40) and the genetic variables were not controlled independently so it is unclear what role each may have played in subtype specification. In contrast to uncultured organoids that have truly EpCAM negative cells, all the cells in pre-stasis HMEC cultures are EpCAM expressing to some extent (Garbe et al., 2012), which may be an adaptation to culture or it may be a selection. In either case, it suggests that we are only transducing EpCAM+ cells. There also may be a retro- and lentivirus transduction bias against luminal cell types (personal communication with Mina Bissell and Curtis Hines). However, we have observed that all lineages of pre-stasis HMEC are transduced to a reasonable extent (a minimum of >40% of each lineage) with a lentivirus that expressed GFP (not shown). Moreover, careful observation of our cultures at each step of the immortalization method showed that the luminal and basal cell types were transduced with the vectors based on antibiotic resistance, and the process of immortalization occurred en mass. Finally, we did not go beyond the non-malignant immortal stage of cancer progression, which best represents the stage of tumor cells in ductal carcinoma *in situ* lesions (Shpitz et al., 1999; Chin et al., 2004). Thus the impact on subtype of additional changes that push non-malignant immortal cell lines all the way to malignancy is still unknown.

### Conflict of interest statement

The authors declare that the research was conducted in the absence of any commercial or financial relationships that could be construed as a potential conflict of interest.
